# Self-Rated Health Status and the Risk of Incident Type 2 Diabetes: A Prospective Cohort Study of Middle-Aged and Older Chinese

**DOI:** 10.3389/ijph.2024.1606401

**Published:** 2024-08-15

**Authors:** Lin Wu, Ruyi Chen, Aiping Sheng, Hongqiang Lou, Xiaowen Wang

**Affiliations:** ^1^ School of Medicine, Jinhua University of Vocational Technology, Jinhua, Zhejiang, China; ^2^ Public Health, Peking University, Beijing, China

**Keywords:** type 2 diabetes, self-rated health status, prospective study, Chinese, cohort

## Abstract

**Objectives:**

Evidence on the relationship between self-rated health status and incident type 2 diabetes (T2DM) in China is scarce. This study aims to examine the prospective association of self-rated health status with the subsequent risk of T2DM among middle-aged and older Chinese subjects.

**Methods:**

Data were obtained from the China Health and Retirement Longitudinal Study of 9844 Chinese individuals aged 45 years or older. Cox proportional hazards models were used to yield hazard ratios (HRs) relating self-rated health status to the 7-year incidence of T2DM, adjusting for conventional risk factors.

**Results:**

Compared to those with very good or good self-rated health, individuals with poor health had a significantly higher risk of developing T2DM in the multivariable-adjusted model [HR = 1.36 (1.07, 1.73)]. Subgroup analysis by sex showed stronger associations in women [HR = 1.53 (1.11, 2.12)]. Interaction analyses indicated that factors such as age, sex, obesity, smoking status, drinking status, history of hypertension and history of dyslipidemia did not modify the association (all *P*-interaction >0.05).

**Conclusion:**

Poor self-rated health status is associated with a higher risk of developing T2DM in middle-aged and older Chinese people.

## Introduction

Type 2 diabetes (T2DM) is a chronic condition characterized by high blood sugar levels due to insulin resistance or inadequate insulin production. It is a significant global health concern [1]. According to the International Diabetes Federation, in 2019, approximately 463 million adults aged 20–79 years were living with diabetes worldwide, and approximately 90% of them had T2DM. The burden of T2DM includes both personal and economic impacts. It affects the quality of life of individuals, increases the risk of premature death, and places a significant economic burden on healthcare systems due to direct medical costs and indirect costs associated with lost productivity and disability [2].

Several risk factors are associated with the development of T2DM. Some of the common traditional risk factors include age, obesity, sedentary lifestyle, and an unhealthy diet [3]. Currently, there is some evidence of associations between self-rated health status and health outcomes. Self-rated health is a subjective measure that asks individuals to assess their own health status. It is often assessed by asking individuals to rate their health as excellent, very good, good, fair, or poor. Research has consistently shown that self-rated health status is a strong predictor of various health outcomes, for example, disease morbidity, mortality, and healthcare utilization [4, 5]. Poor self-rated health has been observed to be associated with a range of conditions such as cardiovascular disease, mental health disorders, and functional limitations [6–8].

Several studies also provided evidence on the relationship between self-rated health status and the risk of incident T2DM [9, 10], showing that poor self-rated health was associated with a higher incidence of T2DM. Participants with poor self-rated health status were more likely to develop T2DM during follow-up, even after adjusting for sociodemographic and health-related factors. However, evidence is scarce in China, particularly among middle-aged and older Chinese, especially considering the challenges posed by the country’s aging population. It is worth mentioning that the association between self-rated health and health outcomes may vary across populations and contexts. Different research studies may have specific findings based on their study design, population characteristics, and other factors.

Therefore, to fill the knowledge gap, the aim of the present study is to investigate the longitudinal association between self-rated health status and the risk of incident T2DM in middle-aged and older Chinese.

## Methods

### Study Participants

**FIGURE 1:**
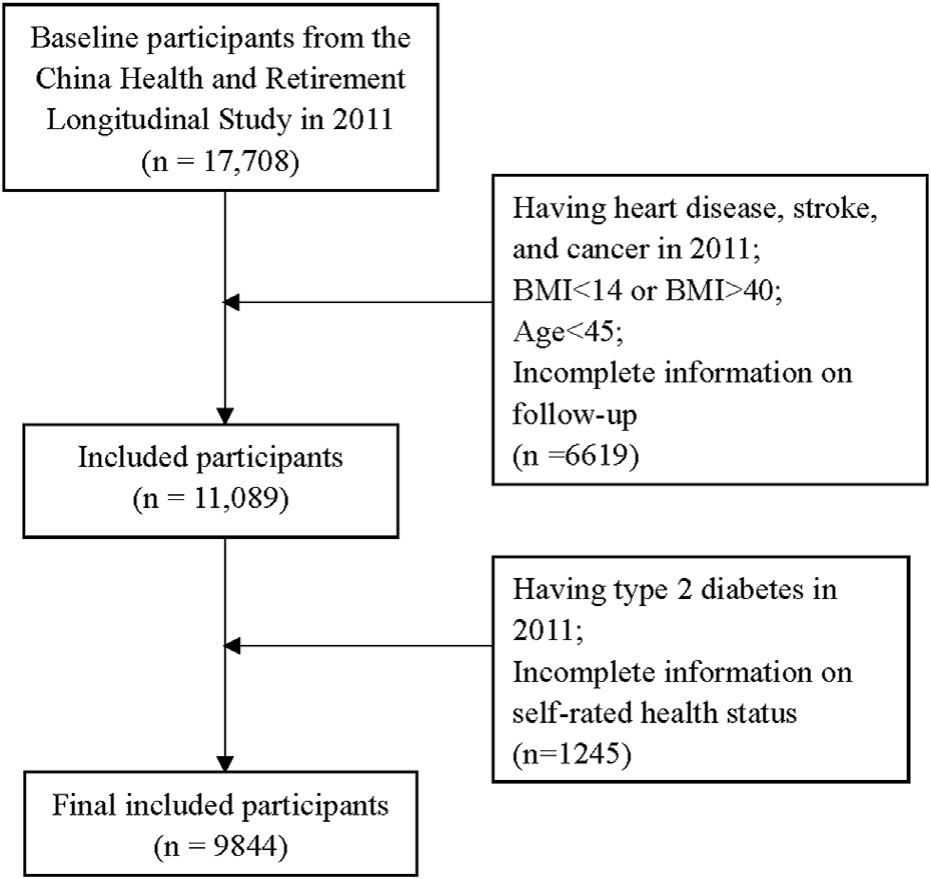
The flow diagram of the participant selection procedure (Zhejiang, China, 2024).

The participants in this study were from the China Health and Retirement Longitudinal Study (CHARLS). Our previous studies have described the design, sampling, and implementation of CHARLS in detail [11–13]. In the present study, the baseline was from 2011 to 2012 and consisted of 17,708 individuals, who were followed every 2 years until 2017–2018. Participants were surveyed via face-to-face interviews and had physical examinations and biochemical tests by well-trained doctors. Those having heart disease, stroke, and cancer at baseline; a body mass index <14 or >40; age <45; and incomplete follow-up information were excluded (n = 6,619). We also excluded those with T2DM at baseline and incomplete information on self-rated health status (n = 1,245). Finally, a total of 9,844 individuals were enrolled in the current analysis (Figure 1).

CHARLS obtained ethical approval from the Biomedical Ethics Review Committee of Peking University (IRB00001052-11015). All participants provided written informed consent.

### Assessment of Self-Rated Health Status

Participants were asked about their self-rated health status with the question, “Would you say your health is excellent, very good, good, fair, or poor?”. This was followed by a similar question, “Would you say your health is very good, good, fair, poor or very poor?”. We combined “excellent,” “very good,” and “good” into “very good or good”; we then combined “poor,” and “very poor” into “poor.”

### Determination of T2DM

Participants were diagnosed with T2DM if they were told that they had T2DM by a doctor, had a fasting blood glucose ≥126 mg/dL or had an HbA1c ≥ 6.5% in 2017–2018 [14, 15]. The validity of the T2DM assessment was established by Wang et al. [15].

### Statistical Analyses

To examine the association between self-rated health status and the risk of T2DM, we estimated hazard ratios (HRs) and 95% confidence intervals (CIs) by using the Cox proportional hazards regression models. People with very good or good self-rated health status were used as the reference group. Model 1 adjusted for sex (male or female) and age (continuous). Model 2 further adjusted for area of residence (urban or rural), education level (less than high school or high school and above), marital status (single or married), BMI (quartiles), moderate physical activity each week (yes or no), current smoker (yes or no), current drinker (yes or no), and sleep (7 and 8 h or other hours). Model 3 was further adjusted for a history of hypertension (yes or no) and a history of dyslipidemia (yes or no). We also conducted subgroup analyses according to sex, age, obesity, smoking status, drinking status, history of hypertension, and history of dyslipidemia, to test whether the association could be modified by these influencing factors. The *p*-value of the interaction was calculated by adding a cross-product term between dichotomous influencing factors (0 and 1) and the three-category variable of self-rated health status variables (0–2). All analyses were performed using SAS version 9.4 (SAS Institute Inc.). *P* values <0.05 were considered statistically significant.

## Results

Table 1 shows the baseline characteristics of 9,844 participants according to their self-rated health status. The numbers of participants who self-rated their health status as very good or good, fair and poor were 2,493 (25.3%), 4,936 (50.1%) and 2,415 (24.5%), respectively. Compared with people who self-rated their health status as very good or good, people with poor self-rated health status appeared to be slightly older and to have a lower BMI. Also, they were more likely to smoke and drink, live in an urban area, be married, have a higher level of education, and have a history of hypertension and hyperlipidemia (Table 1).

**TABLE 1:** Characteristics of participants at baseline according to self-rated health status (Zhejiang, China, 2024).

	Self-rated health status		
	Very good or good (n = 1,493)	Fair (n = 2,936)	Poor (n = 1,415)
Sex			
Men	1,127 (45.2)	2,499 (50.6)	1,396 (57.8)
Women	1,366 (54.8)	1,437 (49.4)	3,019 (42.2)
Age, y	57.9 ± 9.5	58.5 ± 9.4	60.8 ± 9.8
Body mass index, kg/m^2^	23.5 ± 3.4	23.2 ± 3.4	22.6 ± 3.6
HbA1c, %	5.2 ± 0.8	5.2 ± 0.8	5.3 ± 0.9
Fasting blood glucose, mg/dL	109 ± 34	109 ± 35	113 ± 42
Area of residence			
Urban	4,504 (60.3)	2,159 (64.0)	1,841 (76.2)
Rural	989 (39.7)	1,777 (36.0)	574 (23.8)
Level of education			
Less than high school	2,120 (85.0)	4,407 (89.3)	3,297 (95.1)
High school or above	373 (15.0)	529 (10.7)	118 (4.9)
Marital status			
Single	289 (11.6)	571 (11.6)	388 (16.1)
Married	1,204 (88.4)	1,365 (88.4)	2,027 (83.9)
Obesity			
No	1,745 (70.0)	3,538 (71.7)	1,842 (76.3)
Yes	748 (30.0)	1,398 (28.3)	573 (23.7)
Current Smoker			
No	1,618 (64.9)	3,305 (67.0)	1,720 (71.2)
Yes	875 (35.1)	1,631 (33.0)	695 (28.8)
Current drinker			
No	4,451 (58.2)	2,181 (64.4)	1,806 (74.8)
Yes	4,042 (41.8)	1,755 (35.6)	609 (25.2)
Moderate activity/week			
No	2,010 (80.6)	2,006 (81.2)	1,944 (80.5)
Yes	483 (19.4)	930 (18.8)	471 (19.5)
Hypertension			
No	4,168 (87.0)	4,041 (81.9)	1,853 (76.7)
Yes	325 (13.0)	895 (18.1)	562 (23.3)
Hyperlipidemia			
No	2,404 (96.4)	4,655 (94.3)	2,234 (92.5)
Yes	89 (3.6)	281 (5.7)	181 (7.5)

All values are presented as mean (standard deviation) or percentage.

**FIGURE 2:**
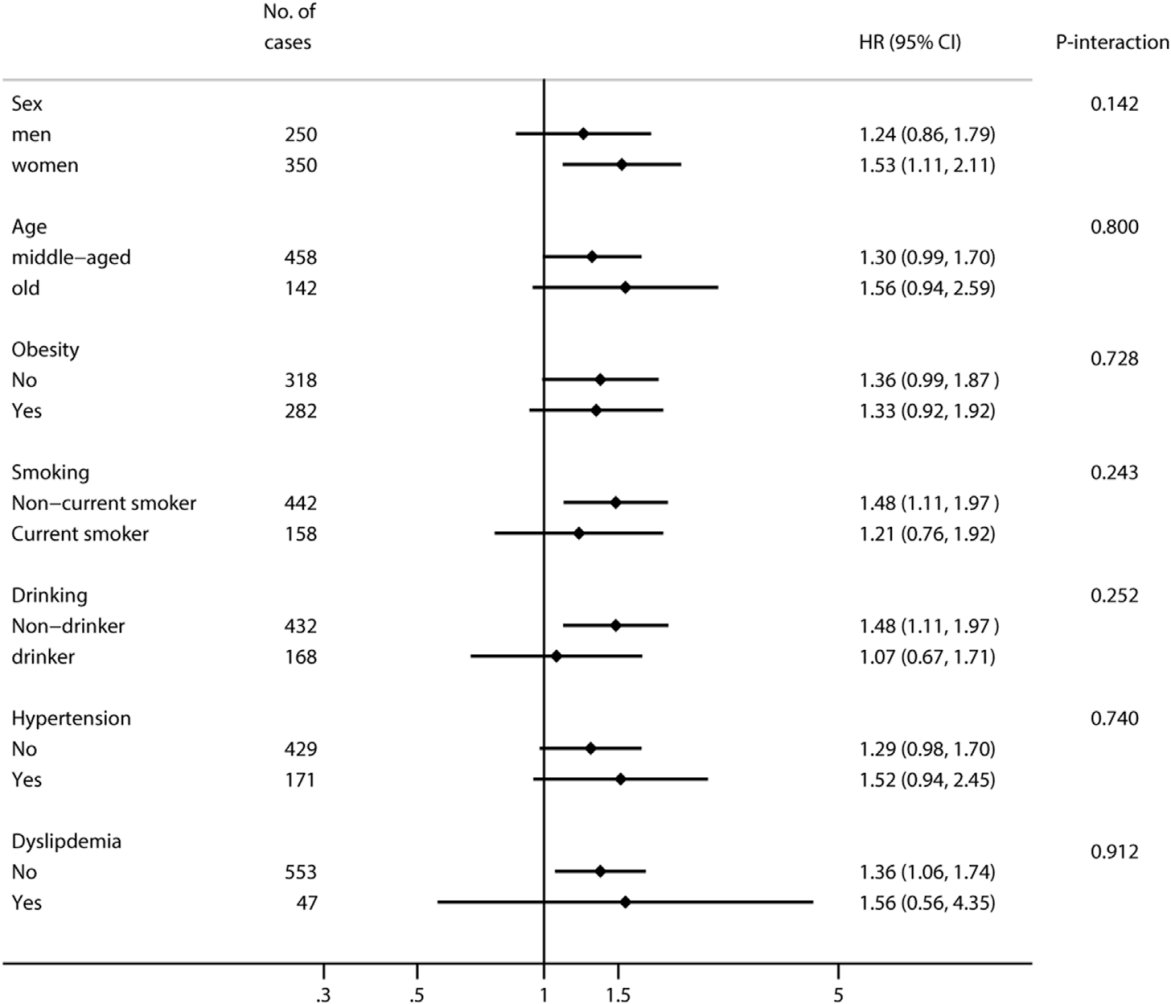
Hazard ratios and 95% confidence intervals for incident type 2 diabetes according to self-rated health status stratified by influencing factors (Zhejiang, China, 2024).

We identified 601 cases of T2DM among 9,844 Individuals during 6 years of follow-up. As is shown in Table 2, compared with people who self-rated their health status as very good or good, people who self-rated their health status as poor had a significantly higher risk of developing T2DM in the age- and sex-adjusted model [HR = 1.38 (1.10, 1.73)]. After adjustment for sex, age, area of residence, marriage, education level, BMI, smoking status, drinking status, moderate physical activity and sleep, the association yielded similar results [HR = 1.42 (1.20, 1.80)]. After further adjustment for history of hypertension and history of dyslipidemia, the association still remained statistically significant [HR = 1.36 (1.07, 1.73)]. Compared with people who self-rated their health status as very good or good, people who self-rated their health status as fair were not significantly associated with developing T2DM (Table 2). Next, we conducted stratified analyses by sex, age, obesity, smoking status, drinking status, history of hypertension and history of dyslipidemia (Figure 2). Subgroup analysis by sex showed that the associations appeared to be evident in women [HR = 1.53 (1.11, 2.12)]. However, interaction analyses indicated that none of the factors modified the association between self-rated health status and the risk of incident T2DM (all P interactions >0.05).

**TABLE 2:** Hazard ratios (HRs) and 95% confidence intervals (CIs) for incident type 2 diabetes according to self-rated health status (Zhejiang, China, 2024).

	Very good or good	Fair	Poor
Number of events	131	295	175
Person-years	16,065.5	31,656.5	15,150.5
Model 1 HR (95% CI)	1.00	1.13 (0.92, 1.39)	1.38 (1.10, 1.73)**
Model 2 HR (95% CI)	1.00	1.15 (0.93, 1.41)	1.42 (1.20, 1.80)**
Model 3 HR (95% CI)	1.00	1.12 (0.91, 1.38)	1.36 (1.07, 1.73)*

Model 1: adjusted for age, and sex.

Model 2: further adjusted for area of residence; marital status; level of education; body mass index; smoking status; drinking status; moderate physical activity; and sleep.

Model 3: further adjusted for history of hypertension and history of hyperlipidemia.

*p values < 0.05; **p values < 0.01.

## Discussion

In this prospective study of middle-aged and older Chinese people, we found that poor self-rated health status, compared with good self-rated health status, was associated with a higher risk of developing T2DM. It remained significant even after adjusting for other risk factors such as age, BMI, and lifestyle factors. This association was also evident in different subgroups of the population.

Several previous studies have examined the relationship between self-rated health and T2DM. Similarly, a case-cohort study from the European Prospective Investigation into Cancer and Nutrition-InterAct Study showed that low self-rated health was associated with a higher risk of T2DM [pooled HR = 1.29 (1.09, 1.53)] [10]. Another cohort study of 250,805 Korean men and women indicated that the HRs for incident T2DM comparing good, fair, and poor or very poor self-rated health with very good self-rated health were 1.20 (0.98–1.48), 1.63 (1.33–1.98), and 1.83 (1.47–2.27), respectively [9]. In addition, self-rated health is associated with quality of life in individuals with T2DM [16, 17]. Those who rate their health as poor or fair often report a lower quality of life across various domains, including physical functioning, mental wellbeing, and social relationships [17]. Self-rated health has also been linked to disease management and control in individuals with T2DM. Studies have shown that individuals who rate their health as poor or fair are more likely to have difficulties managing their diabetes, including poor glycemic control, medication adherence, and engagement in self-care activities [18, 19]. This can lead to complications and poorer health outcomes.

Possible mechanisms underlying the association between self-rated health status and the risk of T2DM warrant discussion. Self-rated health reflects an individual’s overall perception of their health, which may influence their health behaviors. Poor self-rated health may be associated with unhealthy lifestyle behaviors such as a sedentary lifestyle, poor diet quality, smoking, and inadequate physical activity [4]. These behaviors, in turn, increase the risk of developing T2DM. In addition, Self-rated health may be influenced by underlying physiological factors such as hormonal imbalances, metabolic dysregulation, and subclinical diseases [20]. These factors may contribute to both the perception of poor health and an increased risk of developing T2DM. Moreover, poor self-rated health may be associated with increased systemic inflammation and dysregulation of the immune system, as chronic low-grade inflammation and immune dysfunction are known to be involved in the development of insulin resistance and T2DM [21–23]. Further research is needed to better understand the underlying mechanisms and causal relationships between self-rated health and T2DM risk.

Our findings of a stronger association between self-rated health and T2DM risk in women warrant consideration of potential sex differences in health perception and reporting. Research suggests that women may tend to rate their health more accurately and comprehensively compared to men, possibly due to greater health awareness and a higher likelihood of seeking medical care. Women may be more attuned to their health status and more likely to report subjective symptoms and health concerns, leading to a stronger correlation between self-rated health and actual health outcomes [24]. Moreover, societal expectations and roles may influence how women perceive and report their health status, influencing the predictive power of self-rated health in this subgroup. Psychosocial factors such as stress, caregiving responsibilities, and social support networks often differ between men and women and may impact how women perceive and report their health [25, 26]. Future studies exploring these sex-specific differences could further elucidate the mechanisms underlying the observed associations and inform targeted preventive strategies tailored to each sex.

It should be noted that self-rated health is a subjective measure and may be influenced by individual perceptions and experiences. However, it can provide valuable insights into an individual’s perceived health status and its association with T2DM outcomes. Self-rated health encompasses a range of psychosocial factors that can impact health, such as stress, mental health, and social support, which are not always measured in clinical settings. These factors are crucial in understanding an individual’s overall health risk and may explain why SRH is a powerful predictor. Therefore, from a practical standpoint, SRH is an easy and low-cost tool to implement in clinical practice and public health surveys. Its ability to quickly identify individuals at higher risk of adverse health outcomes makes it valuable for early intervention and preventive measures. It is recommended that self-rated health assessments be complemented with clinical measures and evaluations by healthcare professionals for a comprehensive understanding of an individual’s health status and appropriate management of diabetes. Incorporating self-rated health assessments into public health guidelines would support the use of these subjective health measures as valuable tools in population health management. This integration can also facilitate better communication between patients and healthcare providers, fostering a more holistic approach to patient care that considers both objective clinical indicators and the patient’s perceived health status.

This is the first prospective study to show the association between self-rated health status and the risk of incident T2DM in Chinese nationals. We used nationally representative data with a prospective cohort design. However, limitations should be acknowledged. First, the CHARLS study did not conduct a dietary assessment, so we were unable to control for diet as a covariate. Second, this self-reported self-rated health status was subject to misreporting and measurement error, although such measurement errors tended to be non-differential. In addition, although we carefully controlled for several conventional risk factors, residual confounders remain unknown, such as a family history of diabetes. Finally, lifestyle changes (such as smoking status, drinking status, and physical activity changes) may also have an impact on the results.

In conclusion, poor self-rated health status may be associated with a higher risk of developing T2DM. This association highlights the impact of self-perceived health on overall wellbeing and quality of life. The implementation of self-rated health assessments in clinical and public health settings may improve patient outcomes, increase the effectiveness of preventive health strategies, and contribute to a more responsive and patient-centered healthcare system.
